# Bio-organic fertilizers reshape rhizosphere bacterial community and enhance crop productivity in reclaimed soil

**DOI:** 10.3389/fmicb.2025.1713125

**Published:** 2025-12-05

**Authors:** Jing Yang, Xiaomin Lu, Bing Hao, Shimei Wang, Lijuan Kong, Qingqin Shao, Weiping Li, Xingyu Wang, Youzun Xu, Wenge Wu, Jianfei Wang, Lei Yan, Lantian Ren, Gang Chen

**Affiliations:** 1 Anhui Engineering Research Center for Smart Crop Cultivation and Processing Technology, Rice Research Institute, College of Agriculture, Anhui Science and Technology University, Fengyang, China; 2 Rice Research Institute, Anhui Academy of Agricultural Sciences, Hefei, China; 3 Anhui Provincial Agricultural Technology Extension Station, Hefei, China; 4 Anhui Guozhen Environmental Remediation Co., Ltd., Hefei, China; 5 Anhui Agricultural University, Hefei, China

**Keywords:** bio-organic fertilizer, reclaimed soil, crop yield, microbial community, *Sphingomonas*, *Bacillus amyloliquefaciens*

## Abstract

*Bacillus amyloliquefaciens*-modified bio-organic fertilizer (BOF) has shown great potential in improving crop yields and soil quality in degraded or reclaimed soils. However, the underlying microbial mechanisms remain unclear. This study conducted field experiments in reclaimed rice soil to compare the effects of chemical fertilizer (CF), organic fertilizer (OF), and BOF on rice yield and rhizosphere microbial community dynamics. The rice yield of BOF treatment increased by 9.6%, from 7.79 to 8.54 t ha^−1^ compared with CF, and significantly improved critical soil properties by alleviating acidification, as indicated by an increase in pH from 6.10 to 6.65, achieved a 15.7% rise in organic matter accumulation. Available phosphorus increased by 24.5% and available potassium by 14.4%. High-throughput sequencing revealed that bio-organic fertilizer application markedly altered rhizosphere bacterial communities, enriching sulfur-oxidizing taxa and beneficial plant-associated genera including *Sphingomonas*. Quantitative analysis indicated a significant positive correlation between the abundance of *B. amyloliquefaciens* and *Sphingomonas*, and both were associated with improved soil nutrient status and crop performance. Metabolic pathway analysis based on KEGG showed enrichment of the citric acid cycle (1.8-fold) and amino acid biosynthesis (2.3-fold) pathways, which promote nutrient mobilization and microbial interactions. These findings provide new insights into the synergistic interactions between introduced bacteria and native bacterial communities and establish a mechanistic foundation for designing targeted microbial formulations to promote sustainable rice production in improved soils.

## 1 Introduction

As cultivable land becomes scarcer worldwide, reusing reclaimed soil has emerged as a key approach to maintaining food security. In China, the total area of reclaimed soils has exceeded 7.3 million hectares and continues to increase at an annual rate of approximately 3.2% driven by rapid urbanization (Silver et al., 2021). Recent studies indicate that newly reclaimed farmland, particularly sandy soils, exhibits low fertility, poor water retention, and nutrient depletion, severely limiting crop yields during the early stages (Abdelghany et al., 2025). Similarly, studies on reclaimed coastal tidal flats show that soils typically exhibit insufficient organic matter, nitrogen, and phosphorus content, with nutrient pools and ratios significantly lower than those found in conventional farmland (Xie et al., 2021). Therefore, improving the fertility and modifying microbial community structure of reclaimed soils has become an urgent concern in enhancing soil productivity and ensuring sustainable agricultural development.

Although traditional physicochemical amendments, such as lime treatment or fertilization, can effectively regulate soil pH and temporarily enhance nutrient availability, they rarely ensure the long-term stability of soil functions in degraded lands. For instance, research by Villafuerte et al. (2024) indicates that while various organic amendments improve soil physicochemical properties in the short term, the sustainability of these benefits under degraded conditions remains uncertain. In contrast, as the most diverse and functionally active component of agricultural ecosystems, soil microbial communities play a crucial role in nutrient mobilization, organic matter decomposition, plant health regulation, and soil structure stability, directly influencing soil fertility and ecosystem functions (Chauhan et al., 2023). Within microbial populations, bacteria, due to their high abundance, functional diversity, and ability to establish close associations with plant roots, emerge as key drivers of rhizosphere processes. Previous studies have shown that specific bacterial groups (e.g., *Rhizobium*) can enhance nitrogen fixation efficiency in acid-amended soils (Mayhood and Mirza, 2021), highlighting their critical role in maintaining soil productivity. However, existing research has primarily focused on single inoculants or isolated microbial effects, often neglecting the ecological networks and functional interactions that underpin soil remediation. Additionally, efforts to combine microbial community and metabolomics analysis remain limited, resulting in an incomplete understanding of how beneficial inoculants and native microorganisms interact to regulate nutrient cycling. These gaps underscore the necessity of systematic research into how bio-organic fertilizers reshape rhizosphere microbial communities and promote sustainable crop production in amended soils.

The excessive use of chemical fertilizers has caused numerous environmental issues, such as soil acidification, nutrient imbalance, and a decline in soil microbial diversity, all of which crop yields. In many rice-growing regions, nitrogen fertilizer application rates have far exceeded recommended standards, resulting in stagnant yields and long-term soil degradation. Bio-organic fertilizers offer a viable solution by incorporating beneficial microbes that restore soil vitality and boost crop productivity. In fact, extensive research indicates that specific microbial communities, such as *Pseudomonas* and *Bacillus* strains, play a crucial role in enhancing plant productivity, suggesting that bio-organic fertilizers hold potential for promoting the development of such beneficial microbial communities. For example, (Tao et al., 2020) demonstrated that bio-organic fertilizers activate native *Pseudomonas* communities in soil, which form a synergistic relationship with exogenously inoculated *Bacillus* strains, significantly enhancing plant disease resistance and growth. Similarly, (Sultana et al., 2024) found that inoculation with the endophytic *Pseudomonas mosselii* strain PR5 substantially improved rice growth, yield, and nutrient uptake. Although these findings are promising, most studies have focused on conventional farmlands or greenhouse experiments, with limited understanding of the response in reclaimed soils.

Although microbial inoculants have been extensively studied, most research has focused on individual strains, rather than the interactions between introduced microbes and native rhizosphere communities. Emerging evidence from combinatorial screening indicates that rationally formulated mixtures of biostimulants can produce additive or synergistic benefits in plant growth and stress tolerance, including under salt stress, osmotic stress, and temperature stress conditions (Benito et al., 2021). However, comprehensive field evidence is still lacking regarding how BOFs remodel rhizosphere communities and translate into crop performance, particularly in reclaimed soils. Therefore, elucidating the relative contributions of BOF components and their underlying mechanisms is crucial for enhancing the yields of economic crops like rice, promoting sustainable land use, and ensuring food security. At the same time, the historical effects of crops must also be considered. Recent field studies indicate that cropping systems can remodel rhizosphere microbial communities and restore disease suppression capacity, while replenishing depleted native species can guide community assembly toward functional stability (Zhou et al., 2023). For instance, a six-year diversified rotation significantly increased bacterial and fungal diversity (Sun et al., 2024). However, farmers often prioritize rapid economic returns in reclaimed farmland, which makes short-term microbial responses to biostimulants and fertilizers particularly prominent. This underscores the urgent need to elucidate the interaction mechanisms between resident and inoculated microbes over practical timescales and develop targeted biostimulant strategies to rapidly enhance soil quality and crop yields.

Recognizing the importance of functional microorganisms in improving soil quality, we established a field trial that incorporated three distinct fertilization regimes: chemical fertilizer (CF), organic fertilizer (OF), and bio-organic fertilizer (BOF). The trial aimed to examine the impact of bio-organic fertilizer on the soil fertility, crop yield, and underlying microbial mechanisms in reclaimed soil. The field experiment has been managed since 2021 under consistent agronomic practices; however, the present study focuses on the data collected during the 2024 season. The specific objectives of this study are: (1) to evaluate the yield-enhancing effects of bio-organic fertilizer containing *B. amyloliquefaciens* on rice production and soil fertility in reclaimed soils, (2) to investigate how bio-organic fertilizers influence the structure, function, and metabolic dynamics of rhizosphere bacterial communities, including key pathways related to nutrient cycling, and (3) to identify core bacterial taxa and their associated metabolites that are closely related to soil properties and crop yields. The findings of this work are expected to support the design of fertilizer management approaches that not only increase efficiency but also ensure long-term sustainability of grain production in reclaimed agricultural land.

## 2 Materials and methods

### 2.1 Site overview and experimental design

The experimental site was conducted in Guangde City, Anhui Province, China (30°52′41″N, 119°25′16″E). The reclaimed paddy field has been continuously managed since 2021 under uniform cultivation and fertilization practices. However, the present study analyzed samples collected in 2024 only, corresponding to the fourth year of management. The climate is subtropical monsoon, with an annual average temperature of 15.4 °C and precipitation of 1,368 mm. Prior to the trial, the plot had been planted with camphor trees (*Cinnamomum camphora*) for 6–7 years and was converted back to rice paddy fields by the end of 2019. Rice cultivation started in the fertilization treatment areas in 2020. Baseline analysis shows that the reclaimed soil has poor quality, acidity, low organic matter content, and nutrient deficiencies. The soil basic properties were as follows: pH: 6.50, total organic carbon (TOC): 10.90 g/kg, available phosphorus (AP): 18.01 mg/kg, and available potassium (AK): 55.05 mg/kg, total nitrogen (TN): 0.84 g/kg, total phosphorus (TP): 0.60 g/kg, and total potassium (TK): 10.40 g/kg, indicating poor nutrient levels and low soil quality. Additionally, soil electrical conductivity (EC) was 0.38 mS/cm, indicating weak ionic activity. These results confirm that the test site represents poor reclaimed soil, making it suitable for evaluating the effectiveness of bio-organic fertilizer amendment strategies.

**Figure 1:**
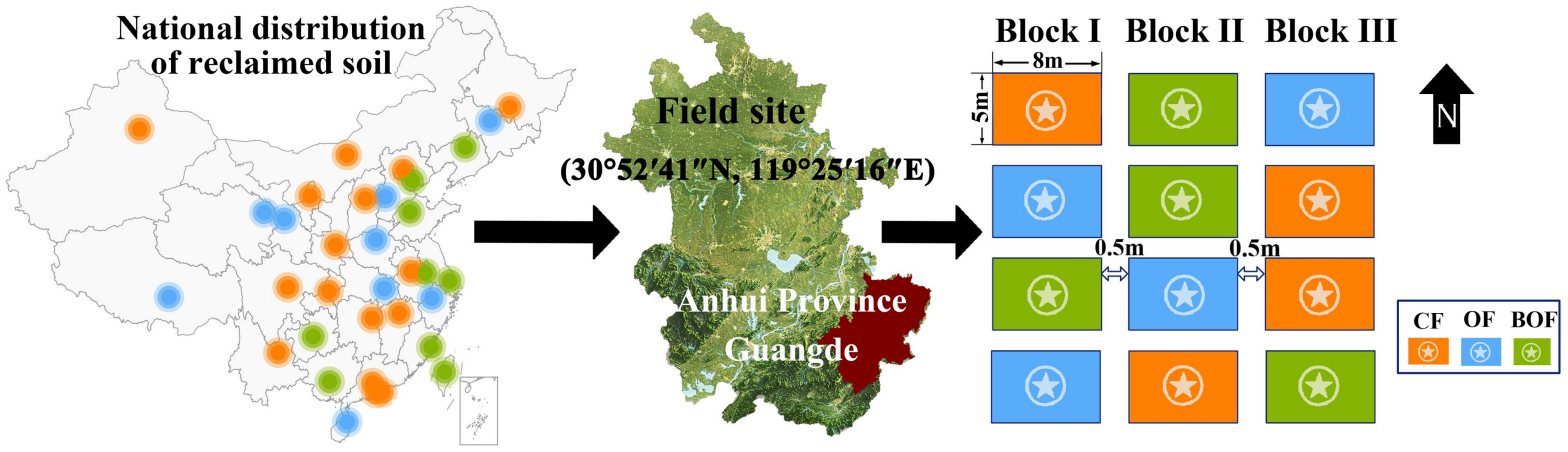
The national distribution map of reclaimed soil and field trial design is shown below. The left panel illustrates the distribution of reclaimed soil in China, categorized by reclamation type: industrial (orange), agricultural (green), and mining (blue). The central panel indicates the experimental site in Guangde city, Anhui province. The right panel shows the randomized block design with three treatment groups: CF (chemical fertilizer), OF (organic fertilizer), and BOF (bio-organic fertilizer with *Bacillus amyloliquefaciens*), each replicated in four plots, measuring 8 × 5 m, with 0.5 m spacing within plots and 1.5 m spacing between plots.

A randomized complete block design was used, with three fertilization treatments: (i) chemical fertilizer (CF), (ii) organic fertilizer (OF), and (iii) bio-organic fertilizer containing *B. amyloliquefaciens* (BOF). Each treatment was replicated four times, with a total experimental area of 667 m^2^ and individual plot dimensions of 8 m × 5 m (Figure 1). The CF treatment applied only chemical fertilizers, including urea (N), calcium superphosphate (P_2_O_5_), and potassium sulfate (K_2_O). The OF treatment was derived from mushroom compost and contained 410 g/kg organic matter, 19.8 g/kg nitrogen, 16.5 g/kg P₂O₅, and 17.3 g/kg K₂O. The bio-organic fertilizer (BOF) was produced by inoculating *B. amyloliquefaciens* at 4.5 × 10^7^ CFU/g dry weight into the same compost substrate.

All treatments were standardized to the same total nutrient input: 240 kg/ha N, 125 kg/ha P_2_O_5_, and 180 kg/ha K_2_O. The application rates of both OF and BOF were 3,300 kg/ha. Fertilizer was applied in three stages: 50% as a basal dose before transplanting, 30% at jointing, and 20% at heading, with a 4:3:3 nutrient distribution. Rice was transplanted in June 2024 and harvested in October 2024.

### 2.2 Soil collection, properties characterization and rice yield measurement

After harvest, five rice plants were randomly selected from each replicate plot. The loose soil was gently shaken off, the roots were immersed in sterile physiological saline, and they were gently shaken to collect tightly adhering rhizosphere soil. The resulting soil suspension was centrifuged at 10,000 × g for 10 min at 4 °C to obtain rhizosphere soil samples (Xiong et al., 2020). The samples were split into two portions: one was snap-frozen in liquid nitrogen and preserved at −80 °C for DNA extraction and sequencing, and the other was subjected to LC–MS-based untargeted metabolomic profiling. In the fourth year of the field experiment, soil samples were collected at two time points (before transplanting and after harvest) to capture changes in soil properties and microbial communities over time. This sampling strategy yielded six sequencing samples per treatment (three randomly selected replicate plots × two time points), which were then used for genomic DNA extraction. Each treatment included four field replicates; however, for sequencing analyses, three replicates were randomly selected to ensure representative sampling. The remaining duplicate samples were retained for agronomic validation. Preliminary analysis of variance indicated no significant differences in yield or soil properties among the four field replicates within each treatment group (*p* > 0.05). Composite soil samples were also collected at the same two time points for further analysis. After removing roots, soil cores were collected from a depth of 0–7 cm. Three subsamples from each treatment group were combined, homogenized, and passed through a 2 mm mesh sieve. These composite soil samples were analyzed for key physicochemical parameters, including soil pH, TOC, TN, TP, TK, AP, AK, and EC.

Rice grain yield was measured at the harvest according to standardized procedures: all plants within the designated harvest area were collected, air-dried, and threshed. A portion of the sample was then dried in a 70 °C oven until a constant weight was reached to determine grain moisture content. Yield data were adjusted to 14% moisture content (Liao et al., 2024).

### 2.3 DNA extraction, 16S rRNA gene amplification and amplicon sequencing

Genomic DNA was isolated from 0.50 g of rhizosphere soil using the Power Lyzer PowerSoil DNA Kit (Qiagen, Germany) according to the manufacturer’s instructions. DNA quality was checked via 1.5% agarose gel electrophoresis, and its purity and concentration were measured with a NanoDrop ND-2000 spectrophotometer (NanoDrop Technologies, USA). For bacterial community analysis, the V3-V4 region of the 16S rRNA gene was amplified with primers 341F (5′-ACTCCTACGGGAGGCAGCAG-3′) and 806R (5′-GGACTACHVGGGTWTCTAAT-3′).

PCR amplification was carried out in a 50 μL system containing 25 μL of 2 × Premix Taq (Takara, Dalian, China), 1 μL of each primer (10 μM), 3 μL of template DNA (20 ng/μL), and nuclease-free water to volume. The thermal program included an initial denaturation at 95 °C for 5 min, followed by 30 cycles of 94 °C for 30 s, 52 °C for 30 s, and 72 °C for 30 s, with a final elongation at 72 °C for 10 min. Amplicons (~400 bp) were excised from a 1.5% agarose gel, purified, and prepared for sequencing. Libraries were generated using the NEBNext Ultra DNA Library Prep Kit (New England Biolabs, USA) with index adapters, and quality was checked on a Qubit 2.0 Fluorometer (Thermo Fisher Scientific) and an Agilent 2,100 Bioanalyzer. Sequencing was performed on an Illumina NovaSeq platform to produce 250 bp paired-end reads. Raw data were filtered with USEARCH, and the clean sequences were deposited in the NCBI SRA under accession PRJNA1332957. Sequencing was conducted by Guangdong Meige Gene Biotechnology Co., Ltd. (China).

### 2.4 Quantitative real-time PCR (qPCR) analysis

Quantitative real-time PCR (qPCR) was performed to determine the absolute abundance of *B. amyloliquefaciens* and *Sphingomonas* in rhizosphere soil samples. DNA extracted from each sample was used as the template for amplification. Species-specific primers targeting the 16S rRNA gene of *B. amyloliquefaciens* and *Sphingomonas* were designed based on conserved regions available in the NCBI database, following protocols adapted from previous studies (Johansson et al., 2014; Zhou et al., 2014). The qPCR reactions were carried out in a 20 μL mixture containing 10 μL of SYBR Premix Ex Taq II (Takara, Japan), 0.4 μL of each primer (10 μM), 2 μL of DNA template, and nuclease-free water to the final volume. Amplifications were conducted on a Bio-Rad CFX96 Real-Time PCR System under the following cycling conditions: 95 °C for 3 min, followed by 40 cycles of 95 °C for 10 s and 60 °C for 30 s. Melting curve analysis was performed from 65 °C to 95 °C to verify the specificity of amplification products.

Standard curves were constructed using ten-fold serial dilutions (10^8^–10^2^ copies μL^−1^) of plasmids containing the corresponding 16S rRNA gene fragments. The amplification efficiency (E) ranged from 90 to 105%, with *R^2^* > 0.99. Gene copy numbers in soil were calculated from the standard curves and normalized to dry soil weight (expressed as target gene copies g^−1^ soil). Each sample was analyzed in triplicate, and no-template controls were included to monitor contamination. The logarithmic values (lg target gene copies g^−1^ soil) were subsequently used for correlation analysis between *B. amyloliquefaciens* and *Sphingomonas* abundances.

### 2.5 LC–MS-based metabolomic analysis and KEGG pathway inference

Rhizosphere soil samples (0.5 g) were freeze-dried and extracted with 80% methanol (v/v) containing internal standards, followed by centrifugation (12,000 × g, 10 min, 4 °C). The supernatant was filtered through a 0.22 μM PTFE membrane and analyzed using a Thermo Fisher Q Exactive™ Orbitrap LC–MS system equipped with an ESI source operating in both positive and negative ionization modes. Chromatographic separation was performed on a C18 reverse-phase column (2.1 × 100 mm, 1.7 μM) under a binary gradient of 0.1% formic acid in water (A) and acetonitrile (B) at 0.3 mL min^−1^. Raw data were processed using Compound Discoverer 3.3 (Thermo Fisher Scientific) for peak alignment, deconvolution, and normalization. The resulting feature table (m/z, retention time, intensity) was imported into MetaboAnalyst 5.0 for multivariate analysis. KEGG pathway enrichment was inferred using the mummichog algorithm (Li et al., 2013) with *Oryza sativa* as the reference organism. Pathways with *p* < 0.05 (Fisher’s exact test) were considered significantly enriched. Functional and biochemical interpretation focused on pathways with the highest pathway impact and biological relevance to nutrient cycling, organic acid metabolism, and redox regulation.

### 2.6 Bioinformatics and data statistical analysis

All datasets were examined for normality and homogeneity of variance prior to statistical analyses. Rice yield and soil physicochemical parameters were analyzed using linear mixed-effects models, with treatment as a fixed effect and block as a random effect. Differences among treatments in soil properties and the relative abundances of dominant bacterial genera were assessed by one-way ANOVA followed by Tukey’s HSD post-hoc tests in SPSS 20.0 (IBM Corp., Armonk, USA). *p*-values were adjusted for multiple comparisons using the Benjamini-Hochberg false discovery rate (FDR) correction. Microbial *α*-diversity (Shannon index) was compared using the non-parametric Wilcoxon rank-sum test implemented in the EASYSTAT package in R (v4.2.2; R Core Team, 2022). *β*-diversity was evaluated based on Bray-Curtis distance matrices and visualized through principal coordinate analysis (PCoA) using the ggplot2 package. Statistical significance among treatments was tested by PERMANOVA (adonis, 999 permutations), following a homogeneity-of-dispersion test (betadisper, vegan package).

Spearman’s rank correlations were calculated to explore associations between microbial community composition and rice yield, while Mantel tests (999 permutations, *p* < 0.05) evaluated relationships between microbial dissimilarities and soil physicochemical variables. Linear discriminant analysis effect size (LEfSe) was applied to identify differentially enriched taxa (LDA score > 2.0, *p* < 0.05). Random forest (rfPermute v2.2) was used to determine the importance of predictor variables, and model significance was verified using the A3 package v1.0.0. Microbial co-occurrence networks were constructed based on Spearman correlations and visualized in Cytoscape v3.9.1. Effect sizes (*R^2^*) and 95% confidence intervals were calculated for key variables.

Functional prediction was performed using 16S rRNA-based genus-level data. Pathway enrichment was inferred against the KEGG pathway database for *Oryza sativa* (as the host crop reference). Significantly enriched pathways were identified using Fisher’s exact test (*p* < 0.05). These predicted pathways provide a computational overview of rhizosphere metabolic processes and should be interpreted as putative functional associations requiring further validation through targeted metabolomics or metagenomic approaches. *Post hoc* power analyses (*α* = 0.05) indicated power = 0.70–0.82 for yield and major soil variables under the observed effect sizes, we limited power for some microbiome comparisons.

## 3 Results

### 3.1 Influence of different fertilization strategies on rice grain yield

**Figure 2:**
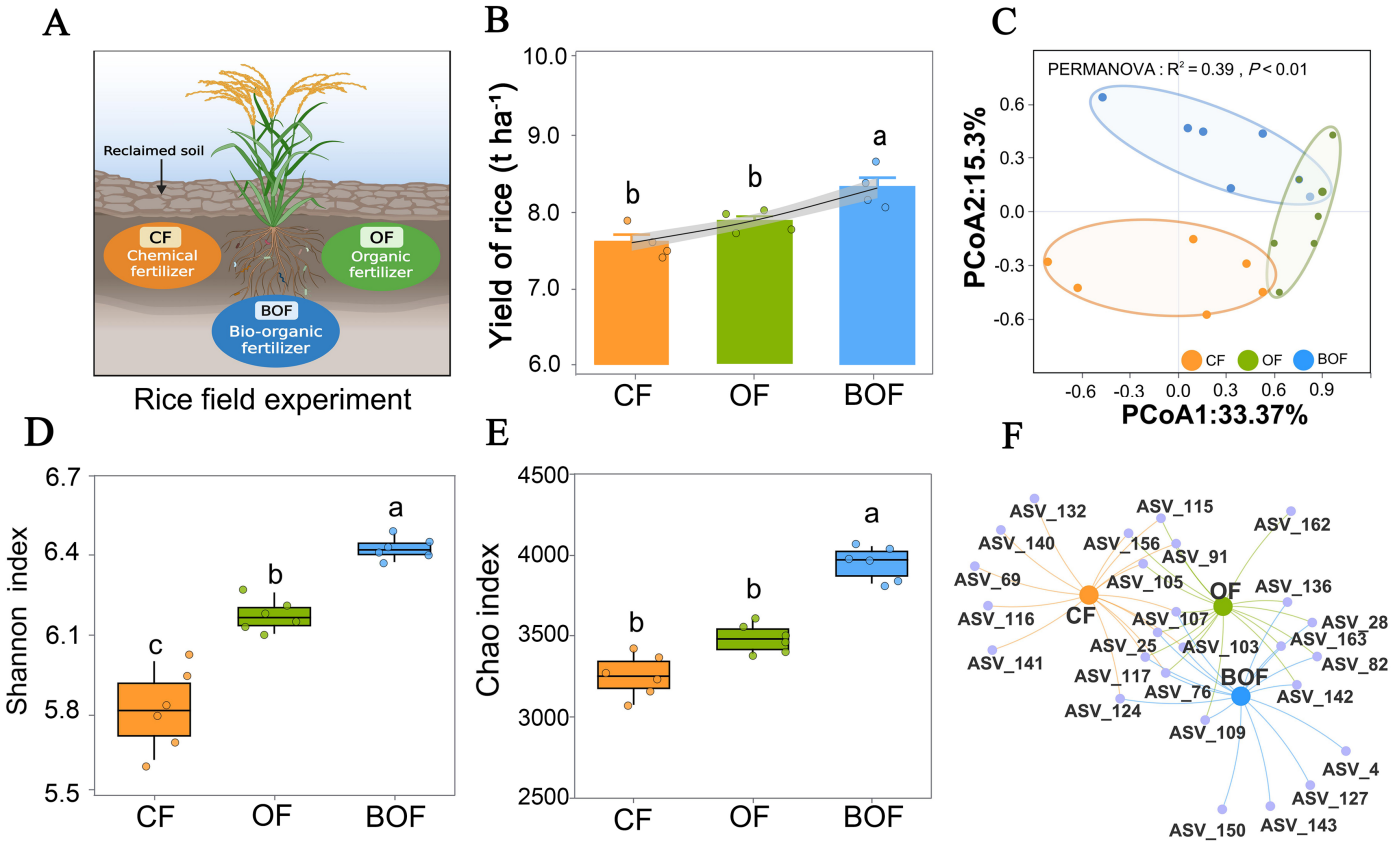
Effects of different fertilization regimes on rice yield and rhizosphere bacterial diversity and community structure in reclaimed soil. **(A)** Schematic diagram of the rice field experiment, with three fertilization treatments: CF (chemical fertilizer), OF (organic fertilizer), and BOF (bio-organic fertilizer supplemented with *Bacillus amyloliquefaciens*). **(B)** Grain yield (t ha^−1^) across fertilization regimes. **(C)** PCoA plot derived from Bray-Curtis dissimilarities. **(D)** Shannon index of rhizosphere bacterial communities under various treatments. **(E)** Chao richness estimates of rhizosphere communities. **(F)** Co-occurrence networks of enriched ASVs, illustrating treatment-specific patterns of microbial assembly. Different lowercase letters above boxplots denote significant treatment differences (Tukey’s HSD, *p* < 0.05).

In the 2024 season, BOF plots showed higher yield and improved soil nutrient status compared with CF and OF plots (Tukey’s HSD test, *p* < 0.05; Figure 2A). Among them, the highest yield was obtained under the BOF treatment, reaching 8.54 t ha^−1^. This yield was 5.8% higher than that under OF (8.07 t ha^−1^) and 9.6% higher than under CF (7.79 t ha^−1^) (Figure 2B). These results indicate that in improved soil, BOF application was more effective than CF or OF alone in enhancing rice yield.

### 3.2 Bacterial community diversity

In terms of *α* diversity, both the Shannon and Chao indices showed significant differences among fertilization treatments (Tukey’s HSD test, *p* < 0.05; Figures 2D,E). BOF exhibited the highest Shannon (5.64) and Chao indices (3,742), while CF treatment had the lowest values (Shannon: 5.54; Chao: 3,215). The OF group exhibited intermediate values (Shannon: 5.59; Chao: 3,458). These results indicate that bio-organic fertilizer significantly enhances the balance of bacterial communities and species richness in remediated soil. PCoA further revealed that BOF samples formed independent clusters from CF and OF (PERMANOVA: *R^2^* = 0.39, *p* < 0.01; Figure 2C), highlighting BOF’s significant restructuring effect on bacterial community composition. Co-occurrence network analysis further indicated that BOF-treated bacterial communities exhibited more complex and unique association patterns than CF and OF treatments (Figure 2F). Notably, key species such as *B. amyloliquefaciens* and *Sulfuricurvum* showed positive correlations with multiple genera, reflecting higher system stability and enhanced ecological functions under BOF. In conclusion, BOF significantly enhanced bacterial diversity and restructured community composition, demonstrating its potential as a sustainable soil fertilization strategy for restoration.

### 3.3 Effects of soil physical and chemical properties

Compared to CF, both OF and BOF significantly improved soil physical and chemical properties, with BOF performing best in most indicators (Table 1). The soil pH under BOF (6.65) was significantly higher than both CF (6.10) and OF (6.34), indicating that BOF effectively alleviated soil acidity. BOF also resulted in the highest TOC (12.61 g/kg), which was 9.0% higher than CF (10.90 g/kg) and 4.7% higher than OF (11.22 g/kg). For nutrient availability, BOF increased AP (24.12 mg/kg) by 34.0% compared to CF (18.01 mg/kg) and 14.6% compared to OF (21.05 mg/kg). Similarly, AK (63.08 mg/kg) under BOF was 14.3% higher than CF (55.15 mg/kg) and 3.5% higher than OF (60.95 mg/kg). In terms of total nutrients, BOF had the highest TN (0.96 g/kg), TP (0.69 g/kg), and TK (12.70 g/kg), all significantly higher than those in CF (0.84, 0.60, and 10.60 g/kg, respectively). The soil EC after BOF was 0.54 mS/cm, higher than CF (0.38 mS/cm) and OF (0.47 mS/cm), reflecting higher ionic strength. Applying BOF improved soil fertility by enhancing organic carbon accumulation, nutrient availability, and total nutrient reserves, while mitigating soil acidity, creating favorable conditions for restoring microbial activity and crop growth.

**Table 1:** Soil physicochemical properties under different fertilization treatments.

Fertilizer treatment	pH	TOC (g/kg)	Available P (mg/kg)	Available K (mg/kg)	Total N (g/kg)	Total P (g/kg)	EC (mS/cm)	Total K (g/kg)
CF	6.10 ± 0.08c	10.90 ± 0.09c	18.01 ± 0.18b	55.15 ± 0.82c	0.84 ± 0.03b	0.60 ± 0.06bc	0.38 ± 0.02b	10.60 ± 0.07bc
OF	6.34 ± 0.03b	11.22 ± 0.05b	21.05 ± 0.17b	60.95 ± 0.66b	0.93 ± 0.01b	0.64 ± 0.05b	0.47 ± 0.03b	11.62 ± 0.03b
BOF	6.65 ± 0.06a	12.61 ± 0.10a	24.12 ± 0.23a	63.08 ± 0.42a	0.96 ± 0.06a	0.69 ± 0.03a	0.54 ± 0.02a	12.70 ± 0.05a

Soil physicochemical properties under different fertilizer treatments are presented as mean ± SD. Distinct lowercase letters (a, b, c) indicate significant differences among treatments based on one-way ANOVA (*p* < 0.05).

### 3.4 Identification of responsive microbial communities

**Figure 3:**
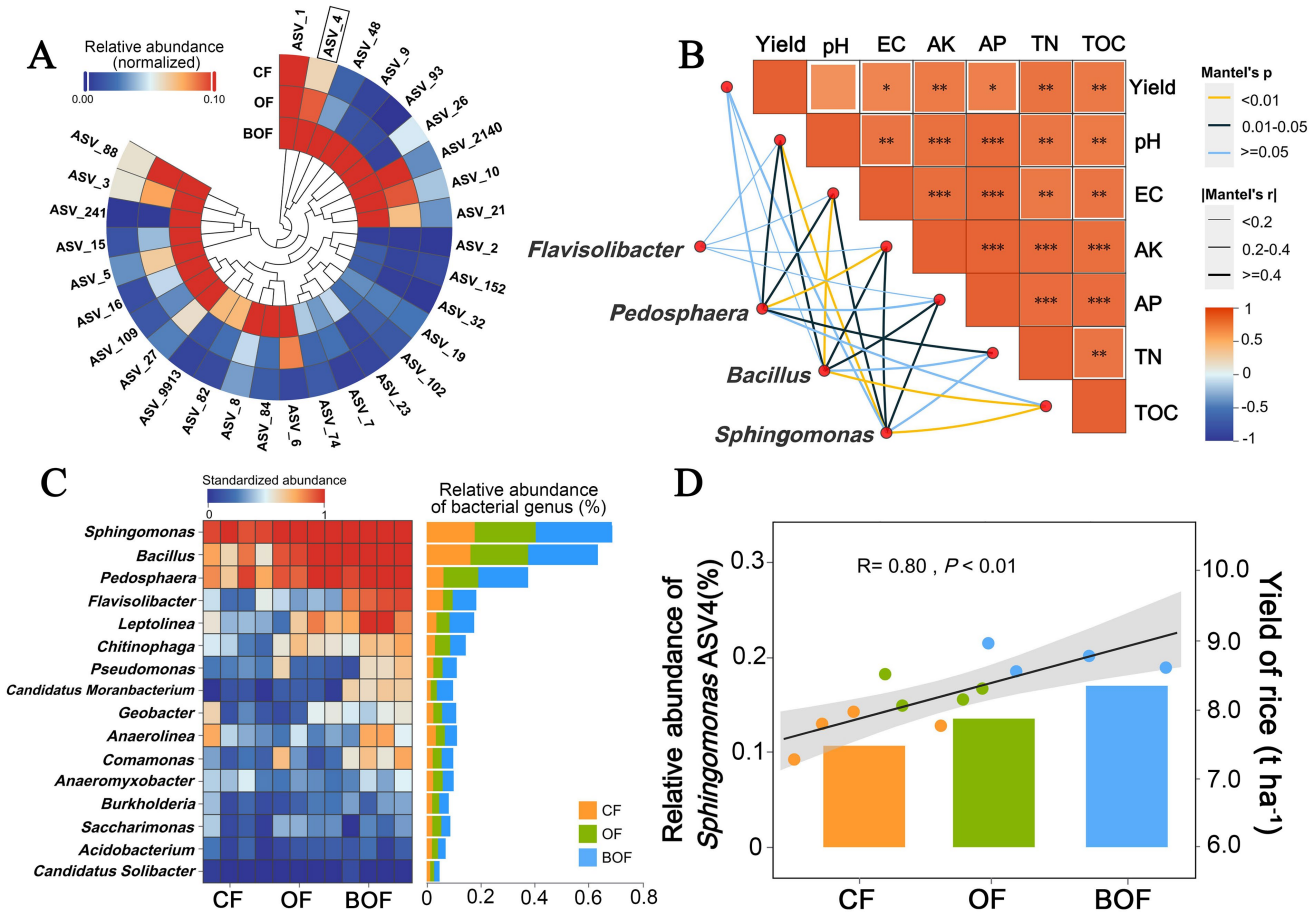
Identification of responsive rhizosphere bacteria under different fertilization treatments and their associations with soil properties and rice yield. **(A)** A circular heatmap shows the differential enrichment of bacterial ASVs under CF, OF, and BOF treatments. Colors represent the relative abundance of each ASV (*n* = 4). **(B)** Association network linking key bacterial genera with soil physicochemical properties and rice yield. The heatmap shows the Mantel correlation coefficients (*r* values) between yield, pH, EC, AK, AP, TN, and TOC. Colored edges indicate significant Mantel correlations (*p < 0.05, **p < 0.01, ***p < 0.001), with edge thickness and color indicating the strength and direction of the correlation. **(C)** Heatmap showing the standardized abundance (left) and total relative abundance (right) of representative bacterial genera across treatment groups. Genera such as *Sphingomonas* and *Bacillus amyloliquefaciens* were enriched under BOF conditions. **(D)** The relative abundance of *Sphingomonas* ASV4 was positively correlated with rice yield (R = 0.80, *p* < 0.01), suggesting its potential contribution to improving soil productivity.

A circular heatmap illustrates the composition and relative abundance patterns of bacterial ASVs under different fertilization treatments. BOF significantly altered the composition of the rhizosphere bacterial community compared with CF and OF (Figure 3A). Genera such as *Sphingomonas*, *Flavisolibacter*, and *Pedosphaera* were enriched under BOF treatment. Given the genus-level resolution of 16S rRNA data and the ecological diversity within each genus, these patterns should be interpreted as putative associations rather than direct evidence of functional roles (Figures 3C,D). The enrichment of these genera under BOF suggests selective stimulation of microbial communities with potential functions for improving soil fertility.

The Mantel test revealed that BOF-treated microbial communities were strongly associated with soil factors such as TOC, AP, and TN (FDR-adjusted *p* < 0.01; Figure 3B). Additionally, regression analysis showed a significant positive correlation between the relative abundance of ASV4 and rice yield (R = 0.80, FDR-corrected *p* < 0.01; Figure 3D). Based on the above results, the observed yield increase under BOF may be partly attributed to the enrichment of specific bacterial groups especially *Sphingomonas*, closely associated with soil nutrient availability and crop performance.

### 3.5 Association of rhizosphere responsive microbes with *Bacillus amyloliquefaciens*

**Figure 4:**
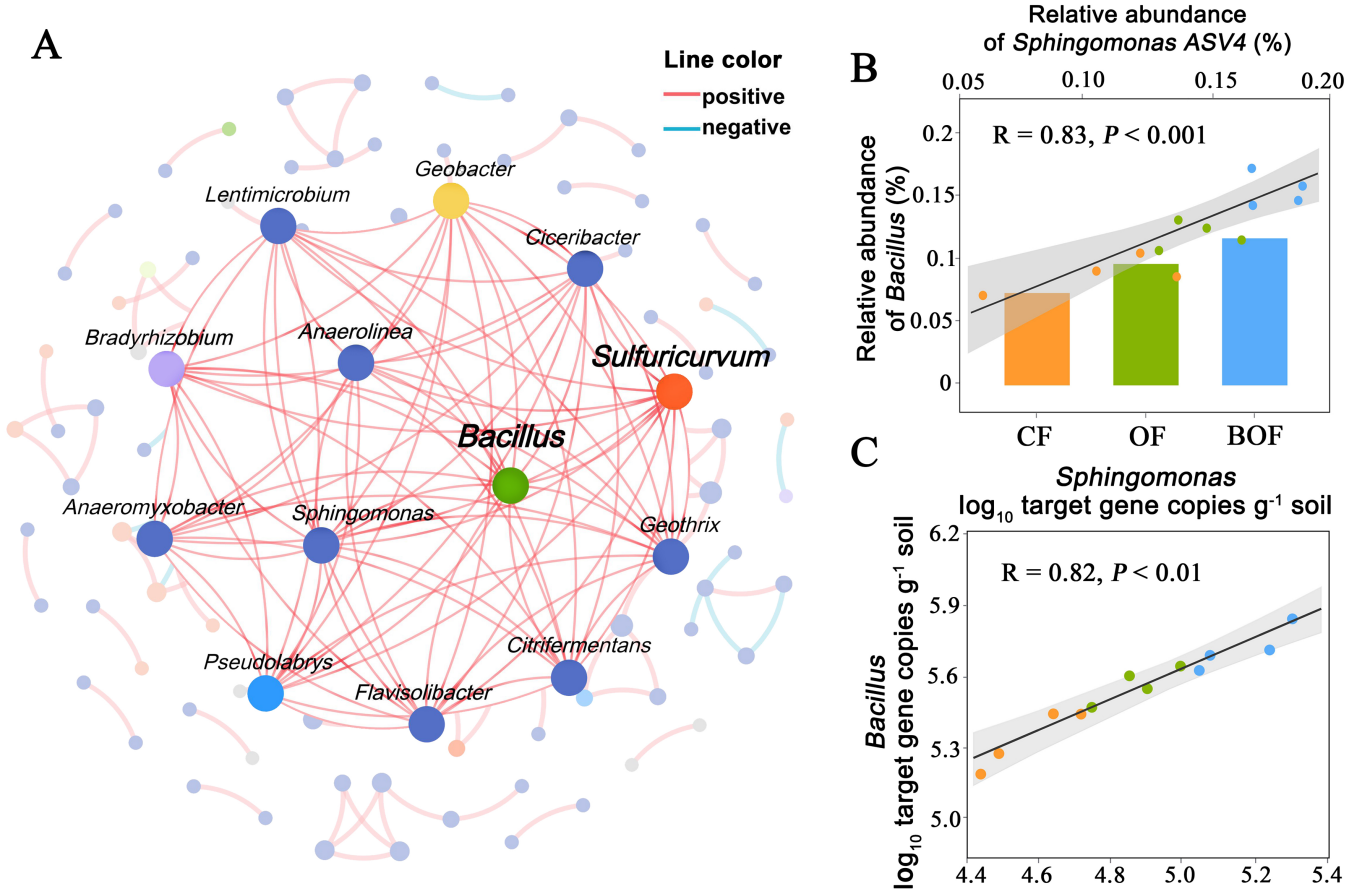
Symbiotic relationships and correlations of *Bacillus amyloliquefaciens*, *Sphingomonas*, and other rhizosphere bacterial groups under different fertilization treatments. **(A)** The coexistence network shows the correlation structure of dominant rhizosphere bacterial genera under all treatment conditions. The network consists of 40 nodes (genus) and 188 edges, with the vast majority (95%) representing positive correlations (red lines) and a few indicating negative correlations (blue lines). Node size reflects the relative abundance of each genus, and node color indicates taxonomic identity. *Bacillus amyloliquefaciens* occupies a central hub position, exhibiting numerous strong positive correlations with genera such as *Sulfuricurvum*, *Sphingomonas*, and *Flavisolibacter*, indicating dense positive co-occurrence patterns under BOF. **(B)** Positive correlation between *Bacillus amyloliquefaciens* and *Sphingomonas* relative abundances across CF, OF, and BOF treatments (*R* = 0.83, *p* < 0.001). **(C)** Absolute quantification of 16S rRNA gene copies confirmed a significant positive correlation between *Bacillus amyloliquefaciens* and *Sphingomonas* in rhizosphere soil (*R* = 0.82, *p* < 0.01).

Genus-level co-occurrence network analysis was used to explore the associations between the inoculated strain *B. amyloliquefaciens* and indigenous rhizosphere bacteria. The resulting network revealed several dominant modules, with *B. amyloliquefaciens* and *Sulfuricurvum* occupying central positions. More than 90% of significant associations represented positive correlations (Figure 4A). The relatively understudied genus *Sulfuricurvum* exhibited strong connectivity with *B. amyloliquefaciens*, suggesting a potential ecological role in nutrient-depleted reclaimed soils, consistent with its reported sulfur-oxidizing activity in similar environments (Figure 4B), which likely reflects co-occurrence within similar rhizosphere niches or shared responses to BOF-induced soil changes rather than verified metabolic synergy. This association was further supported by qPCR absolute quantification, which showed a significant positive correlation between their gene copy numbers (R = 0.82, *p* < 0.01; Figure 4C). These parallel patterns may result from similar ecological preferences or independent responses to shifts in soil nutrient status and pH following BOF application. Although these findings indicate ecological compatibility, they do not constitute direct evidence of functional or metabolic interaction.

Overall, BOF application was associated with the successful establishment of inoculated *B. amyloliquefaciens* and two complementary ecological outcomes: (i) enrichment of known beneficial genera such as *Sphingomonas*, consistent with potential compatibility rather than confirmed synergism; and (ii) recruitment of novel genera such as *Sulfuricurvum*, revealing additional ecological pathways potentially involved in rhizosphere restructuring. This dual perspective suggests that BOF not only reinforces established plant-microbe associations but also facilitates the emergence of previously underrecognized microbial groups, thereby contributing to network stability and improved soil functionality.

### 3.6 Cooperative interactions between rhizosphere microbes and metabolite accumulation

**Figure 5:**
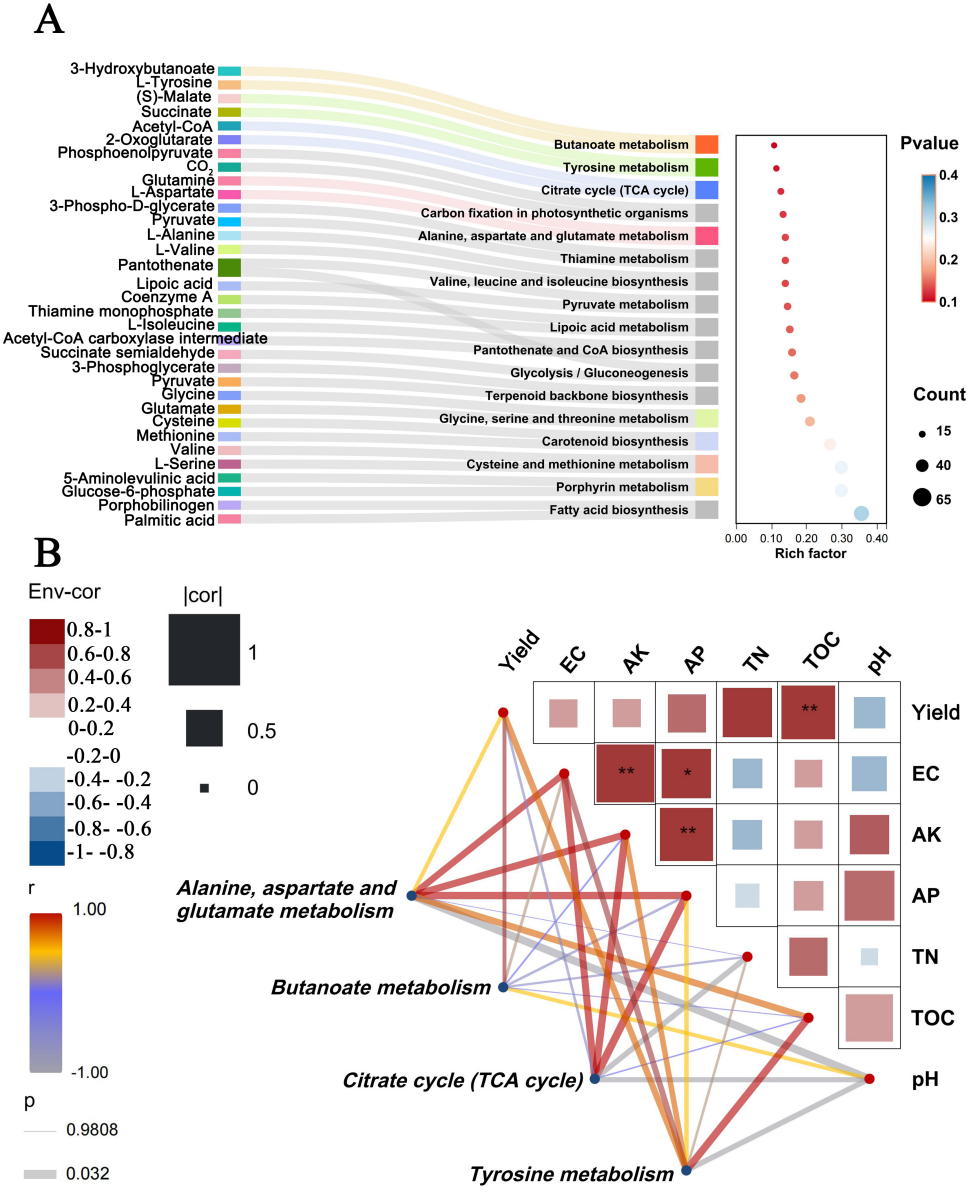
Functional enrichment of microbial metabolic pathways and their correlation with rice yield and soil properties. **(A)** KEGG pathway enrichment analysis of microbial genes affected by bio-organic fertilizer treatment. The Sankey diagram shows enriched pathways associated with various metabolic functions, including the Citrate cycle (TCA cycle), butanoate metabolism, tyrosine metabolism, and alanine, aspartate, and glutamate metabolism. The bubble chart displays the corresponding pathway enrichment values based on enrichment factors, gene counts, and adjusted *p*-values (color gradient). **(B)** Correlation analysis between selected enriched pathways and environmental variables (including rice yield and soil physicochemical parameters (pH, EC, AK, AP, TN, TOC)). The upper**-**right matrix displays Mantel’s *r* correlation coefficients and significance levels (*p < 0.05, **p < 0.01, ***p < 0.001). The lower left section displays a bipartite network connecting pathways and environmental factors; line thickness and color represent the strength and direction of the Pearson correlation coefficient (*r*), while transparency indicates the significance level. Pathways significantly correlated with yield and soil fertility parameters, particularly amino acid metabolism (alanine, aspartate, and glutamate) and the citric acid cycle, are highlighted as key microbial functional processes underlying the efficacy of bio-organic fertilizers.

Untargeted LC–MS metabolomic profiling revealed marked differences in soil metabolite composition among the three fertilization treatments. A total of 254 metabolites were annotated, including organic acids, amino acids, and other low-molecular-weight compounds associated with nutrient transformation and microbial metabolism, indicating that bio-organic fertilization established a distinct rhizosphere biochemical profile. Pathway enrichment analysis identified citric, malic, and succinic acids, pyruvate, glutamine, aspartate, and tyrosine as key contributors (Figure 5A). KEGG annotation demonstrated significant enrichment of pathways related to carbon and nitrogen turnover, including the citrate cycle (TCA cycle), alanine - aspartate - glutamate metabolism, butanoate metabolism, and tyrosine metabolism. Higher rich factors and lower *p* values under BOF suggest enhanced microbial energy metabolism and amino acid biosynthesis in the rhizosphere. Correlation analysis further revealed strong positive relationships between these pathways and soil fertility indicators (Figure 5B). The TCA cycle and alanine - aspartate - glutamate metabolism correlated significantly with total nitrogen, available phosphorus, and yield (*p* < 0.05), whereas butanoate and tyrosine metabolism showed positive associations with available potassium and total organic carbon. These patterns indicate that bio-organic fertilizer stimulated microbial metabolic activity and promoted nutrient cycling through the synthesis and turnover of key organic intermediates.

Collectively, these findings demonstrate that functional pathway enrichment under BOF aligns with compositional shifts in soil metabolites. The co-occurrence of abundant organic and amino acid metabolites with improved soil fertility suggests that BOF application fosters an active and nutrient-efficient rhizosphere environment, ultimately enhancing rice productivity in reclaimed soil.

## 4 Discussion

### 4.1 Impacts of bio-organic fertilization on soil nutrients and crop productivity in reclaimed land

Compared with CF and OF, the BOF treatment substantially improved soil fertility in reclaimed paddy soil (Table 1). These improvements were accompanied by higher bacterial diversity, enrichment of *Bacillus* and *Sphingomonas*, and enhanced central carbon and amino acid metabolism, suggesting that microbial restructuring played a central role in regulating soil chemical properties.

Soil pH. Although LC–MS analysis indicated increased organic acid turnover under BOF, the overall soil pH rose from 6.10 in CF to 6.65 in BOF. This trend can be explained by the combined effects of base-cation inputs and enhanced cation exchange capacity through organic matter humification, which increase base saturation and buffering capacity (Das et al., 2023). In addition, ligand complexation of Al and Fe reduces exchangeable acidity and releases Ca and Mg, while microbial oxidation of organic anions consumes protons, resulting in a net increase in bulk pH. TOC and total nutrients. BOF increased TOC, TN, TP, and TK mainly through additional organic-matter inputs and microbial immobilization–mineralization processes that improved nutrient retention as soil pH and CEC increased (He et al., 2024; Zhang et al., 2025). Available P. The higher level of AP under BOF likely resulted from the combined action of organic-acid chelation of Fe/Al-bound phosphate and enhanced microbial P solubilization, both favored by the moderate pH rise (Deng et al., 2022). Available K. Greater AK availability was associated with microbial weathering of soil minerals and stronger K^+^ retention on exchange sites due to improved soil aggregation and organic matter accumulation (Yadav et al., 2021). Electrical conductivity. A slight increase in EC reflected the higher ionic strength associated with greater nutrient availability, remaining within a non-saline range beneficial for crop growth (Jensen et al., 2024).

Collectively, these findings indicate that BOF promotes a metabolically active and better-buffered rhizosphere environment through synergistic interactions between organic inputs and beneficial microbes. This microbiome-driven improvement in soil properties provides a coherent explanation for the observed enhancement in fertility and yield of rice in reclaimed soil systems.

### 4.2 Impacts of applying bio-organic fertilizers on the structure and diversity of microbial communities in the rhizosphere

Reclaimed soils are typically characterized by low organic matter content, poor structure, and depleted microbial communities, which limit microbial colonization and ecological functioning (Liu et al., 2021; Liu et al., 2023a). Under these conditions, the application of bio-organic fertilizers (BOFs) significantly enhances microbial *α*-diversity. This improvement can be attributed to the additional organic matter and nutrients provided by BOFs, which enhanced soil porosity, moisture retention, and buffering capacity, creating more heterogeneous and stable ecological niches for microorganisms (Cui et al., 2023; Hamamoto et al., 2022). These improved microhabitats allowed a wider range of microbial taxa to establish and coexist, resulting in higher species richness and evenness. With respect to *β*-diversity, reclaimed soils often exhibited homogenized microbial communities dominated by a few opportunistic taxa due to nutrient scarcity and weak ecological interactions. The introduction of BOFs altered this dynamic by diversifying nutrient resources and modifying soil conditions, thereby reshaping community assembly processes and reducing the uniformity often observed under sole chemical fertilization (Kong et al., 2023; Pan et al., 2025). This promoted greater differentiation among microbial communities across treatments, reflecting a shift toward greater ecological complexity and stability (Gul et al., 2023; Jensen et al., 2024).

Taken together, these findings suggested that BOFs improve soil fertility but also stimulate the ecological succession of microbial communities in reclaimed soils. Given their initially impoverished microbial status, reclaimed soils offered a unique context for further research into how functional microbial groups and soil structural improvements interact to enhance diversity and ecosystem functionality. This laid the groundwork for subsequent investigations into key microbial taxa.

### 4.3 The role of bio-organic fertilizer in stimulating core microorganisms

Co-occurrence network analysis in this study highlighted that BOF application stimulated several core microbial taxa in the rice rhizosphere, with *Sphingomonas* being most consistently enriched. Although not traditionally categorized as a plant growth-promoting microorganism (PGPM), previous research has shown its involvement in phosphorus solubilization, auxin production, and biofilm formation, functions that contribute to plant growth under stress conditions (Deng et al., 2022; Guo et al., 2024). The enrichment of *Sphingomonas* under BOF treatment, suggested that this genus may serve as a context-dependent PGPM in reclaimed soils.

*Sphingomonas* was found to be consistently enriched in soils treated with BOF. Previous studies (Liu et al., 2025; Martins et al., 2023) have reported members of this genus as potential plant growth-promoting bacteria under certain environmental conditions. In the context of this study, its enrichment may simply reflect an adaptive response to BOF-induced soil changes, and its specific functional role in rice systems remains to be verified in future work. Their co-occurrence suggested a functional consortium that stabilizes the rhizosphere environment and enhances nutrient cycling, representing a potential synergistic mechanism underlying the beneficial effects of BOF on soil improvement. In addition to these dominant genera, the activation of rarer taxa such as *Sulfuricurvum* highlighted the involvement of overlooked microbial groups in maintaining sulfur cycling and redox balance (Jiang et al., 2021; Liu et al., 2023b). However, while these network-based associations provided valuable insights, the causal links between core microbial taxa, metabolite production, and plant performance remained to be verified.

Taken together, these findings suggested that BOF stimulated core microbial groups with potential plant growth*-*promoting roles, either directly or through interaction with *B. amyloliquefaciens*. Importantly, the ecological functions of these taxa were linked to metabolic shifts in the rhizosphere, which warrants further examination of BOF-induced metabolomic changes to clarify how microbial activation translates into improved nutrient availability, soil stability, and crop yield (Vandenkoornhuyse et al., 2015).

### 4.4 LC**–**MS-based metabolomic insights and functional interpretation

Untargeted LC–MS metabolomic profiling revealed distinct shifts in rhizosphere metabolic composition following BOF application. These changes were consistent with a reorganization of soil metabolic pathways rather than a simple supplementation of external nutrients. Such alterations are particularly valuable in reclaimed soils characterized by poor fertility and depleted microbial activity. Pathways associated with amino acid metabolism, nitrogen cycling, and organic acid biosynthesis were significantly enriched, indicating accelerated nutrient turnover and improved microbial carbon use efficiency (Marschmann et al., 2024; Wang et al., 2022). The increased levels of citric and malic acids suggested enhanced phosphorus mobilization, a key process in nutrient-limited reclaimed soils. Elevated concentrations of amino acid-related metabolites (e.g., glutamine, asparagine) indicated improved nitrogen assimilation, while higher abundances of phytohormone-related compounds such as indoleacetic acid (IAA) implied stimulated root growth and rhizosphere activity (Louca et al., 2016). Additionally, enriched sulfur-related metabolites and KEGG sulfur metabolism pathways reflected intensified redox regulation, potentially contributing to pH buffering and chemical stabilization under waterlogged conditions (Geng et al., 2023).

Collectively, these metabolomic findings indicate that BOF application was associated with a shift from nutrient-deficient, microbially impoverished soils toward metabolically active and functionally resilient systems. By promoting nutrient mobilization, supporting root development, and stabilizing redox processes, BOF-induced metabolic enhancement may provide a biochemical basis for the observed yield increases 6.53 to 18.03%, consistent with prior bio-organic fertilization studies (Zhang et al., 2024). The concurrent enrichment of *B. amyloliquefaciens* and *Sphingomonas* further suggests that these microorganisms could participate in nutrient-related transformations such as sulfur and nitrogen cycling, thereby improving soil chemical stability. Overall, these results support the view that BOF acts not only as a nutrient source but also as an ecological catalyst that stimulates native microbial communities. Future multi-omics studies integrating metabolomics, metagenomics, and transcriptomics will be essential to validate the underlying metabolic exchanges and microbial signaling processes driving these associations.

### 4.5 Taxonomic resolution, functional inference, and study limitations

The associations identified in this study were derived from co-occurrence and correlation analyses based on 16S rRNA gene sequencing. For instance, the positive association observed between and *Sphingomonas* may reflect shared environmental preferences, parallel responses to BOF-induced soil changes, or other indirect ecological processes rather than verified metabolic cooperation (Berry and Widder, 2014; Faust and Raes, 2012). Therefore, all relationships discussed here should be regarded as putative associations until experimentally validated.

This study focused exclusively on the bacterial component of the rhizosphere, as inferred from 16S rRNA gene sequencing. Fungal communities-which typically comprise a major proportion of soil microbial biomass-were not analyzed. Yet, fungi, particularly arbuscular mycorrhizal fungi (AMF) and saprophytic taxa, play essential roles in nutrient turnover, phosphorus acquisition, and organic matter decomposition (Bender et al., 2016). Consequently, the reported “rhizosphere microbial community” patterns represent a bacteria-centered and partial perspective of the soil microbiome. Future research integrating ITS-based fungal profiling, mycorrhizal quantification, and LC–MS-based metabolomics will be critical for developing a more comprehensive understanding of how BOF influences the entire rhizosphere ecosystem.

The taxonomic resolution of the present dataset-restricted to the genus level-also limits the precision of functional and mechanistic interpretation. Genera such as *Sphingomonas*, *Flavisolibacter*, and *Pedosphaera* encompass diverse species with markedly different ecological roles, as also observed in microbial communities exhibiting taxonomic-functional decoupling, ranging from pathogenic to plant-beneficial strains (Louca et al., 2018; Liu et al., 2024). Hence, genus-level correlations with soil pH, nutrient availability, or crop yield should be viewed as indicative rather than mechanistic. Higher-resolution metagenomic and metatranscriptomic analyses will be required to resolve species-or strain-specific contributions to BOF-mediated soil improvement.

**Figure 6:**
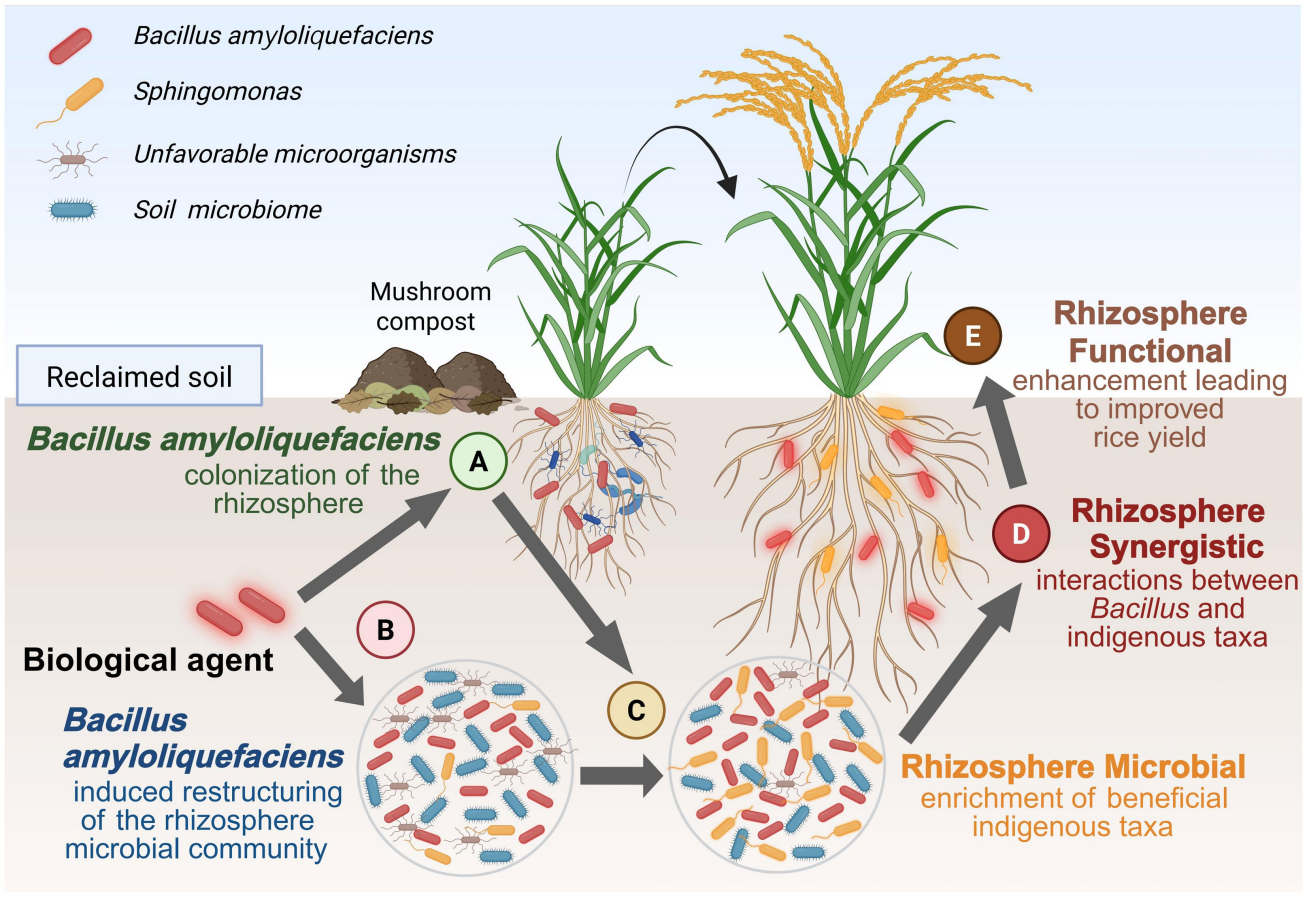
Hypothesis-level conceptual model illustrating putative associations among BOF application, rhizosphere community shifts, soil nutrient status, and rice yield based on single-year (2024) observations. Arrows denote hypothesized associations rather than proven causal relationships. Taxa are presented at the genus level, and functional roles are not inferred at species or strain level. **(A)** *Bacillus amyloliquefaciens* colonizes the rhizosphere, enhancing bacterial interactions. **(B)** *Bacillus amyloliquefaciens* influences bacterial communities, particularly reshaping the structure of rhizosphere bacterial communities. **(C)** Induced rhizosphere bacterial community reorganization promotes enrichment of beneficial bacterial taxa. **(D)** Synergistic interactions between *Bacillus amyloliquefaciens* and bacterial taxa stimulate formation of more stable and beneficial bacterial networks. **(E)** Enhanced rhizosphere bacterial community function improves rice growth and yield in reclaimed soils, promoting plant health and productivity.

Finally, this investigation represents a single-site, single-year field study conducted within a broader multi-year management framework. Temporal variability and long-term responses were beyond the scope of the present dataset, and the functional inference derived from 16S rRNA data remains inherently limited. Future long-term monitoring including seasonal and interannual variations would help clarify temporal stability of BOF effects. Thus, the conceptual framework presented in Figure 6 should be interpreted as a hypothesis-level model, illustrating plausible ecological linkages rather than confirmed causal pathways. Future multi-year, multi-omics investigations are needed to validate these mechanisms and to evaluate the persistence and generality of BOF effects across environmental and temporal gradients.

## 5 Conclusion

In reclaimed paddy soil, the application of bio-organic fertilizer (BOF) containing *B. amyloliquefaciens* was associated with higher rice yield, improved soil fertility, and distinct alterations in rhizosphere bacterial communities during the 2024 growing season. As schematically illustrated in Figure 6, we propose a hypothesis-level conceptual framework linking BOF application, rhizosphere restructuring, and enhanced soil nutrient dynamics; this framework is illustrative and requires further validation. The present findings reflect associations observed during a single growing season, and additional multi-year investigations are needed to evaluate their long-term stability and agronomic significance. Nevertheless, these preliminary results provide meaningful guidance for the rational design of targeted bacterial formulations that integrate exogenous inoculants with indigenous bacterial communities to support sustainable and resilient fertilizer strategies in reclaimed soils. Future multi-year and multi-domain investigations, incorporating both bacterial and fungal community analyses, will be crucial to fully elucidate the ecological mechanisms by which BOF influences soil fertility and crop productivity.

## Data Availability

The datasets presented in this study can be found in online repositories. The names of the repository/repositories and accession number(s) can be found in the article/supplementary material.
